# Phosphorus Localization and Its Involvement in the Formation of Concentrated Uranium in the Renal Proximal Tubules of Rats Exposed to Uranyl Acetate

**DOI:** 10.3390/ijms20194677

**Published:** 2019-09-20

**Authors:** Shino Homma-Takeda, Chiya Numako, Keisuke Kitahara, Takanori Yoshida, Masakazu Oikawa, Yasuko Terada, Toshiaki Kokubo, Yoshiya Shimada

**Affiliations:** 1 National Institute of Radiological Sciences, National Institutes for Quantum and Radiological Science and Technology, 4-9-1 Anagawa, Chiba 263-8555, Japan; keisuke_kitahara@hotmail.com (K.K.); yoshida.takanori@qst.go.jp (T.Y.); oikawa.masakazu@qst.go.jp (M.O.); kokubo.toshiaki@qst.go.jp (T.K.); shimada.yoshiya@qst.go.jp (Y.S.); 2 Institute for Quantum Life Science, National Institutes for Quantum and Radiological Science and Technology, 4-9-1 Anagawa, Chiba 263-8555, Japan; 3 Graduate School of Science, Chiba University, Yayoi-cho, Chiba 263-8522, Japan; numako@chiba-u.jp; 4 Graduate School of Science and Engineering, Chiba University, Yayoi-cho, Chiba 263-8522, Japan; 5 Japan Synchrotron Radiation Research Institute, Mikazuki, Hyogo 679-5198, Japan; yterada@spring8.or.jp

**Keywords:** uranium, kidney, distribution, μ-PIXE (particle-induced X-ray emission with microprobe), SR-μXRF (X-ray fluorescence analysis with microprobe), μXAFS (X-ray absorption fine-structure with microprobe)

## Abstract

Although the kidneys comprise a critical target of uranium exposure, the dynamics of renal uranium distribution have remained obscure. Uranium is considered to function physiologically in the form of uranyl ions that have high affinity for phosphate groups. The present study applied microbeam-based elemental analysis to precisely determine the distribution of phosphorus and uranium in the kidneys of male Wistar rats exposed to uranium. One day after a single subcutaneous injection of uranyl acetate (2 mg/kg), areas of concentrated phosphorus were scattered in the S3 segments of the proximal tubule of the kidneys, whereas the S3 segments in control rats and in rats given a lower dose of uranium (0.5 mg/kg) contained phosphorus without concentrated phosphorus. Areas with concentrated phosphorus contained uranium 4- to 14-fold more than the mean uranium concentration (126–472 vs. 33.1 ± 4.6 μg/g). The chemical form of uranium in the concentrated phosphorus examined by XAFS was uranium (VI), suggesting that the interaction of uranyl ions with the phosphate groups of biomolecules could be involved in the formation of uranium concentration in the proximal tubules of kidneys in rats exposed to uranium.

## 1. Introduction

Uranium is a naturally occurring radioactive heavy metal that can cause nephrotoxicity [1,2]. The applications of uranium in the nuclear industry and in military projects have led to increasing public concern over its health effects. The chronic ingestion of naturally occurring uranium in contaminated groundwater results in increases in urinary markers associated with renal tubular injury [3]. Uranium-induced renal toxicity is characterized by the induction of tubular lesions in the S3 segments of the proximal tubule (S3 segments), the distal portion of the proximal tubule [2,4]. We previously investigated the cellular dynamics of uranium distribution in the S3 segments of rat kidneys during acute renal toxicity using high-energy X-ray fluorescence elemental analysis with a microprobe and high-energy synchrotron radiation (SR-μXRF) [5]. High concentrations of uranium in microregions were observed near the nuclei of the epithelium of the S3 segments and detectable during recovery. The mechanistic details of the formation of uranium concentration in toxic target sites should be determined to reduce renal uranium toxicity and find an effective method of decorporating accumulated uranium from the kidneys.

Uranium is considered to act as uranyl ions (UO_2_^2+^) in aqueous environments and can be toxic to living organisms [1]. Uranyl acetate (UA), as well as uranyl nitrate, is commonly used in toxicological studies (for example, [4,6,7,8]). Uranyl ions have high affinity for phosphates [6,9]. Studies of cultured cells in vitro have shown that uranium compounds precipitate in the cytoplasmic compartment as uranyl phosphate needles after exposure to toxic concentrations (400–2000 μM) of uranium [7,8,10]. However, the renal distribution of phosphorus during uranium toxicity remains obscure. High-energy SR-μXRF is excellent for detecting uranium at trace levels in tissues but is not good at detection of light elements, such as phosphorus, potassium, and calcium, that can now be measured using particle-induced X-ray emission with microprobe (μ-PIXE) analysis [11,12,13].

We previously reported that a single subcutaneous injection of 2 mg kg^−^^1^ (body weight) of uranyl acetate (UA) into male Wistar rats caused renal lesions in the S3 segments [14]. Apoptotic cells increased in the S3 segments on post-administration day 2, damaged tubules without a brush border and cells reached a maximum on day 8, and then the damaged tubules were partly renewed on day 15. The present study used μ-PIXE to determine the renal distribution of phosphorus in situ in the rat model. Colocalized phosphorus and uranium in the S3 segments was also assessed using μ-PIXE and SR-μXRF, and the chemical form of the colocalized uranium in microregions was assessed by X-ray absorption fine-structure with microprobe (μXAFS).

## 2. Results and Discussion

### 2.1. Phosphorus Distribution in Rat Kidneys after Uranium Exposure

**Figure 1:**
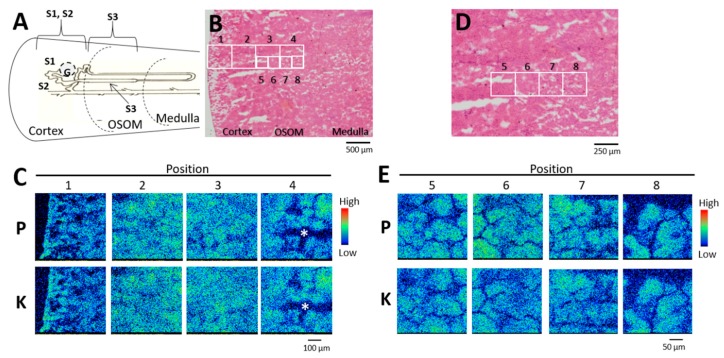
Phosphorus and potassium distribution in control kidney. (**A**) Nephron unit distribution. G, glomeruli; S1, S2, and S3, segments of proximal tubule. (**B**,**D**) Serial sections stained with hematoxylin and eosin. (**C**) Elemental imaging (scanned area, 500 μm × 500 μm; integrated current, 0.2 μC; beam size, 1 μm × 1 μm) in boxed areas 1–4 in (**B**). (**E**) Elemental imaging (scanned area, 250 μm × 250 μm; integrated current, 0.2 μC; beam size, 1 μm × 1 μm) in boxed areas 5–8 in (**D**). P, phosphorus; K, potassium. *Artificial crack. The periphery of the renal cortex is on the left side of all images.

**Figure 2:**
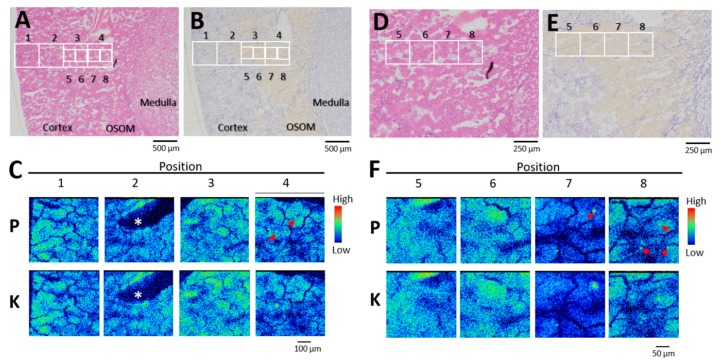
Phosphorus and potassium distribution in kidney on day 1 post-administration of uranyl acetate (UA) (2 mg kg^−1^). (**A**,**D**) Serial sections stained with hematoxylin and eosin. (**B**,**E**) Serial sections stained for glutamine synthetase. Yellow tubules, S3 segments of the proximal tubule. (**C**) Elemental imaging (scanned area, 500 μm × 500 μm; integrated current, 0.2 μC; beam size, 1 μm × 1 μm) in boxed areas 1–4 (A and B). (**F**) Elemental imaging (scanned area, 250 μm × 250 μm; integrated current, 0.2 μC; beam size, 1 μm × 1 μm) of boxed areas 5–8 (D and E). P, phosphorus; K, potassium. Arrows, phosphorus concentrated areas. *Artificial crack. The periphery of the renal cortex is on the left side of all images.

**Figure 3:**
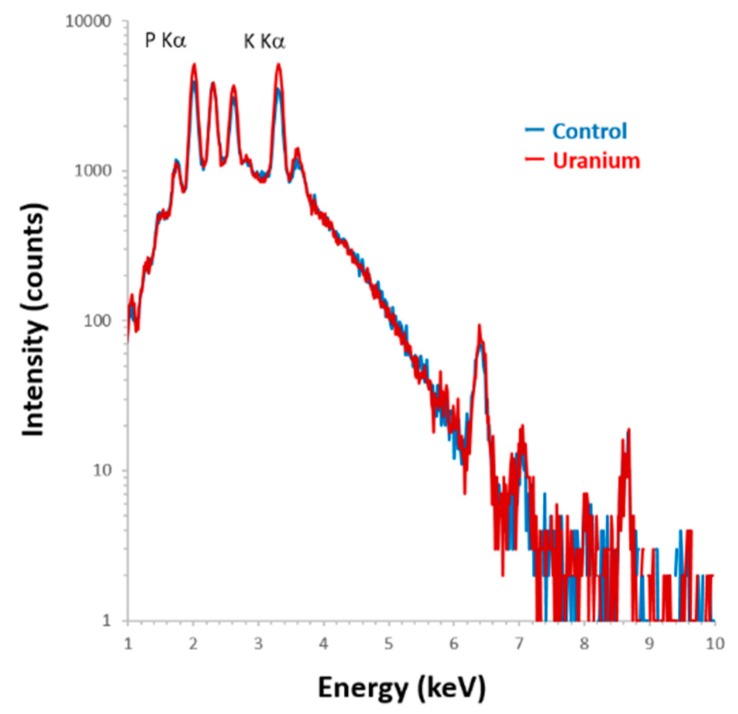
Particle-induced X-ray emission (PIXE) spectra obtained from scanning at position 7 in Figure 1E and Figure 1F. Blue line, control; red line, uranium-treated; scanning area, 250 μm × 250 μm, integrated current: 0.2 μC, beam size: 1 μm ×1 μm with Si (Li) detector.

Phosphorus distribution in rat kidneys was analyzed on days 1 and 3 (onset of toxicity) after administration of uranyl acetate (UA) (2 mg kg^−^^1^). The areas covering the outer cortex (OC; segments S1 and S2, upper part of the proximal tubules), the inner cortex (IC), and the outer strip of the outer medulla (OSOM; S3 segments) were analyzed using elemental imaging (Figure 1A,B). Phosphorus, potassium, and calcium were equally distributed throughout the OC (Figure 1C, positions 1 and 2), the IC (Figure 1C, position 3), and the OSOM (Figure 1C, position 4) without site-specificity in control rats, which was unlike the site-specific renal uranium distribution in the IC and OSOM [14,15]. A comparison of hematoxylin and eosin (HE)-stained serial sections (Figure 1D) using high-resolution phosphorus imaging (Figure 1E) confirmed that the proximal tubules in the IC and OSOM contained phosphorus, but it was not localized. By contrast, phosphorus concentrations were found in the OSOM on day 1 post-uranium administration (Figure 2C). A comparison of serial sections that were immunostained for glutamine synthetase (Figure 2E) and analyzed by high-resolution phosphorus imaging showed scattered phosphorus and potassium colocalization in the epithelium of most S3 segments of the proximal tubule (Figure 2F). Representative examples of spectra obtained from segments of the S3 region (position 7) are shown in Figure 3. The levels of phosphorus and potassium in the S3 region of uranium-treated rats were 1.4-fold and 1.6-fold higher than those of the controls without phosphorus and potassium concentrations.

**Figure 4:**
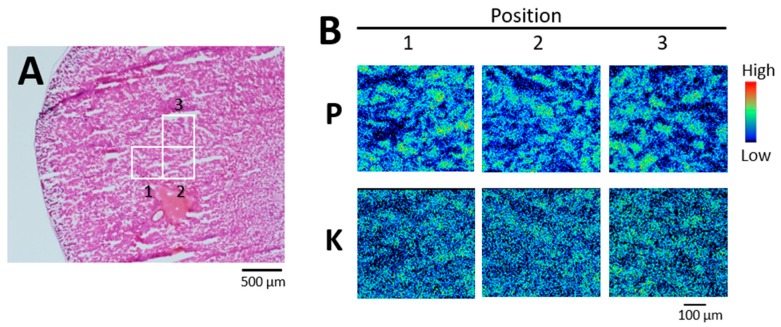
Phosphorus and potassium distribution in kidney on day 1 post-administration of UA (0.5 mg kg^−1^). (**A**) Serial section stained with hematoxylin and eosin. (**B**) Elemental imaging (scanned area, 500 μm × 500 μm; integrated current, 0.2 μC; beam size, 1 μm × 1 μm) of boxed areas 1–3 (**A**). P, phosphorus; K, potassium. The periphery of the renal cortex is on the left side of all images.

We analyzed two to six 500 × 500 μm fields in the IC and OSOM of each rat. Areas of concentrated phosphorus were detected in two of three rats on day 1 post-administration, and in all three rats on day 3 post-administration. By contrast, concentrated phosphorus was not observed in any of three rats given 0.5 mg kg^−^^1^ UA on day 1 post-administration (Figure 4) or the control rats.

### 2.2. Colocalization of Uranium in Areas of Concentrated Phosphorus in the S3 Segments of the Proximal Tubule

**Figure 5:**
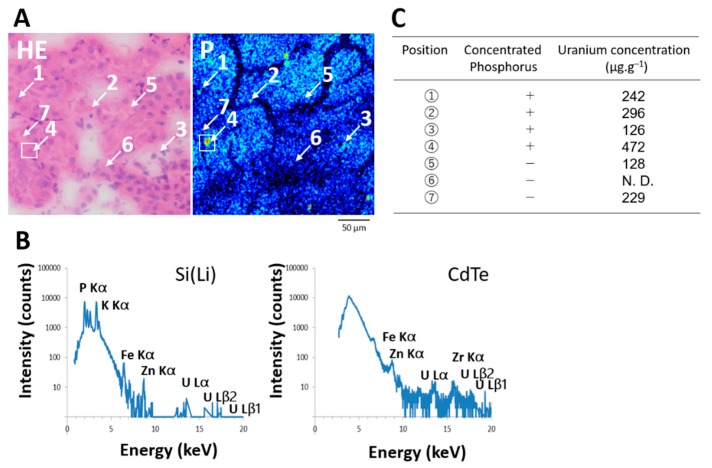
Quantitative local analysis of uranium in areas with and without concentrated phosphorus in the S3 segments of the proximal tubules on day 1 post-administration of UA (2 mg kg^−1^). (**A**), Analytical points for PIXE with microprobe (μ-PIXE) spot analysis (scanned area, 500 μm × 500 μm; integrated current, 0.2 μC; beam size, 1 μm × 1 μm). Points 1–4 and 5–7 with and without concentrated phosphorus, respectively. HE, hematoxylin and eosin. P, phosphorus imaging (scanned area, 250 μm × 250 μm; integrated current, 0.2 μC; beam size, 1 μm × 1 μm). (**B**), μ-PIXE spectra of boxed area (**A**) (scanned area, 30 μm × 30 μm; integrated current, 0.2 μC; beam size, 1 μm × 1 μm) with Si (Li) and CdTe detectors. Zr was from materials of CdTe detector. (**C**), Uranium values in areas (**A**) with and without concentrated phosphorus. N. D., not detected.

The μ-PIXE spectra showed that areas of concentrated phosphorus in the S3 segments at day one after UA administration contained uranium (Figure 5A,B).

We applied μ-PIXE spot analysis to measure uranium values in randomly selected areas of the S3 segments with and without concentrated phosphorus. Uranium values in areas of concentrated phosphorus were 4- to 14-fold higher than the mean renal values (126–472 vs. 33.1 ± 4.6 μg·g^−1^), whereas the highest value in the areas without concentrated phosphorus was 7-fold higher than the mean renal values (229 vs. 33.1 ± 4.6 μg·g^−1^) **(**Figure 5C).

**Figure 6:**
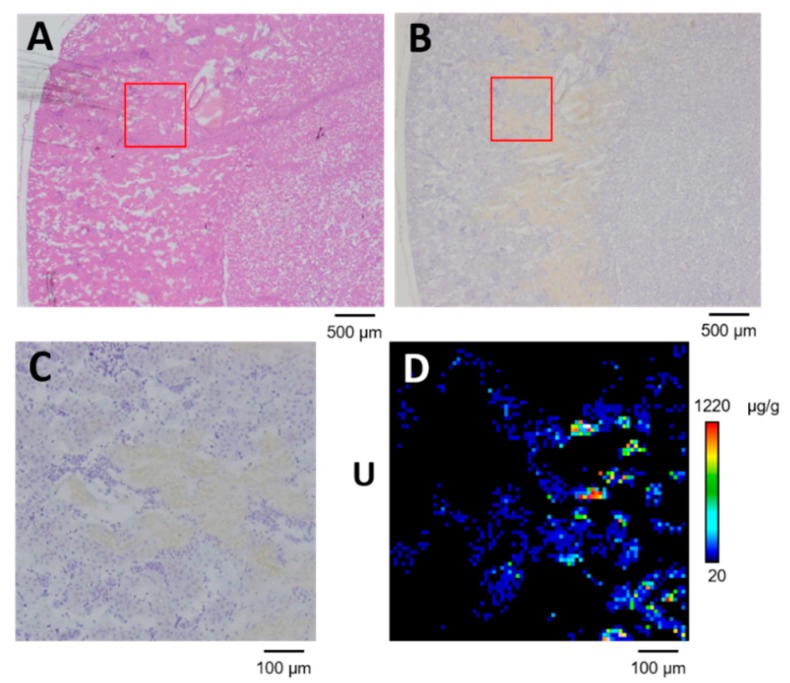
Uranium distribution in OSOM of kidney with scattered concentrated phosphorus on day 1 post-administration of UA (2 mg kg^−1^). (**A**) Serial section stained with hematoxylin and eosin. (**B**) Serial section immunostained for glutamine synthetase. (**C**) High-resolution image of boxed area in (**A**) and (**B**). (**D**) Uranium imaging (75 × 75 steps at 10 μm per step; beam size, 1 μm × 1 μm). The periphery of the renal cortex is on the left side of all images.

We used SR-µXRF imaging to confirm uranium distribution in serial sections of the renal specimen that was analyzed using μ-PIXE spot analysis (100 μm from the section shown in Figure 5). Uranium was distributed in the S3 segments of the proximal tubule (Figure 6). Up to 1220 μg·g^−1^ of uranium accumulated in the S3 segments, which was more than the amount in areas with concentrated phosphorus. These findings indicated different types of concentrated uranium in the proximal tubules after exposure. The mechanism of formation of concentrated uranium in the proximal tubules might vary according to the elemental composition.

### 2.3. Chemical Form of Uranium in Areas of Phosphorus Accumulation

**Figure 7:**
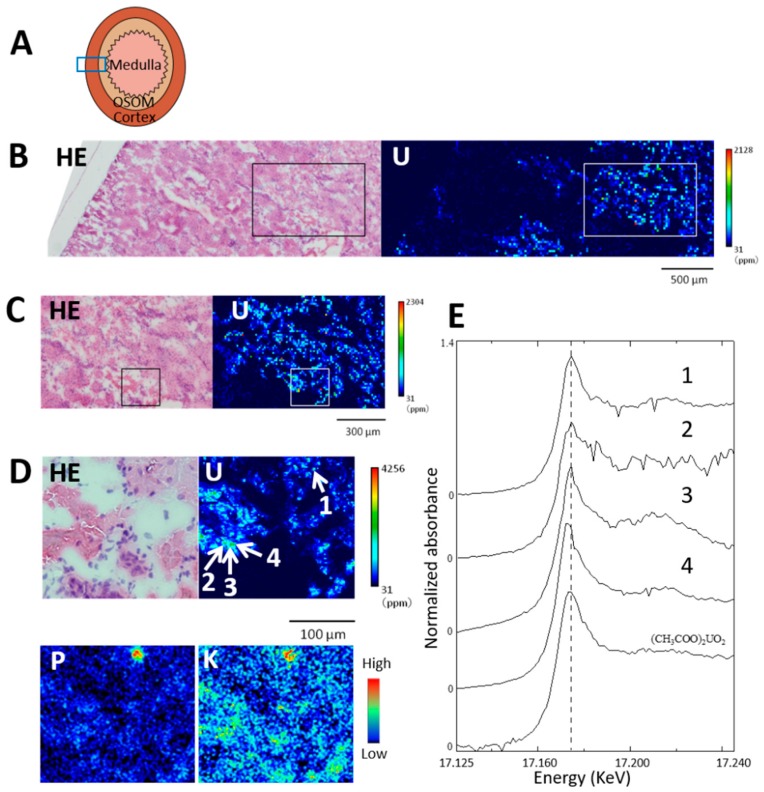
Uranium distribution in kidney and uranium LIII-edge XANES (X-ray absorption near edge structure) spectra of concentrated uranium in the S3 segments of the proximal tubule. Renal section on day 3 after administration of UA (2 mg kg^−1^). (**A**) Analyzed area of renal specimen. (**B**) Uranium imaging (150 × 50 steps at 20 μm per step; beam size, 1 μm × 1 μm). HE, hematoxylin and eosin. (**C**) High-resolution uranium imaging of boxed area in (B) (100 × 60 steps at 10 μm per step; beam size, 1 μm × 1 μm). (**D**) High-resolution uranium (110 × 100 steps at 2 μm per step; beam size 1 μm × 1 μm) and μ-PIXE imaging of phosphorus and potassium in boxed area in (C). Point 1, concentrated phosphorus; points 2, 3, and 4, without concentrated phosphorus. Uranium levels at points 1, 2, 3, and 4 were 2919, 938, 1980, and 2627 μg g^−^^1^, respectively. The periphery of the renal cortex is on the left side of all images. Mean renal uranium concentration was 24.1 μg g^−^^1^. (**E**) LIII-edge XANES spectra of concentrated uranium in the S3 segments of the proximal tubule; graphs 1, 2, 3, and 4 are for points 1, 2, 3, and 4 in panel (**D**).

The chemical form of uranium in areas of renal specimens with and without concentrated phosphorus was examined on day 3 post-administration (Figure 7). The area analyzed in Figure 7B corresponds to the diagram in Figure 7A and extends from the OSOM to the periphery of the renal cortex. Uranium was distributed in the IC and OSOM to a maximum that exceeded 2000 μg·g^−1^. Most renal tubules in the boxed area in Figure 7C were the S3 segments of the proximal tubule and contained uranium. The boxed area in Figure 6C corresponds to the area visualized by phosphorus and potassium imaging (Figure 7D). Four points (1–4) in Figure 7D were assessed using elemental analysis: point 1, located in the epithelium of the S3 segments with concentrated phosphorus as well as concentrated potassium; points 2, 3, and 4 were located in the epithelium of the same tubule without concentrated phosphorus. The uranium values at points 1, 2, 3, and 4 were 2919, 938, 1980, and 2627 μg·g^−1^, respectively.

The U LIII-edge XANES spectrum of point 1 with concentrated phosphorus was similar to that of UA in terms of the spectral shape and the energy position of the peak (17.1746 keV, Figure 7E). On the other hand, both uranyl acetate-like and reduced form-like XANES spectra of U LIII-edge were found in areas without concentrated phosphorus; the XANES spectrum of point 4 showed a slightly negative chemical shift from 17.1746 to 17.1734 keV with a slightly narrower peak width, whereas the spectral shapes and energy positions of the peak tops of points 2 and 3 were close to those of UA.

Our previous XAFS study of uranium showed that the XANES spectrum of uranium absorbed into cellulose phosphate under biological conditions (pH 7.4) was similar to that of UA, uranium (VI) [16], indicating that interactions between uranyl ions (VI) and phosphorus could explain the uranium colocalization in the S3 segments. Most of the uranium that accumulated in the kidneys was uranium (VI), but some had an edge that slightly shifted towards lower energy, meaning that uranium was reduced after administration [16]. The reduction of uranium (VI) to (V) or (IV) results in a slight shift of the edge jump at 1–2 eV and a slight narrower peak [17,18], indicating that the proximal tubules without concentrated phosphorus could contain chemical forms of uranium that varied between (VI) and a reduced form. Further examinations with several reduced standards and improved spectral quality are needed to clarify the formation of the reduced form of uranium.

Most of the areas of concentrated phosphorus determined herein contained potassium, but not calcium (Figure 3). Mineralization of calcium with phosphorus might not be a major event in the formation of phosphorus concentration at the initial phase of uranium exposure. Inorganic interactions between phosphorus and potassium are also unlikely to be involved in that process. Uranyl ions have high affinity for phosphate groups on biomolecules, and their interactions have been analyzed [6,9,19]. For example, osteopontin is key to the regulation of mineralization in the proximal tubules of kidneys, and it can bind uranyl ions [6,20,21]. The phosphorylation of osteopontin results in increases in uranium binding [6]. Therefore, interactions between uranium and phosphorus in biomolecules might be involved in the mechanism of uranium accumulation rather than inorganic interactions between uranium and phosphorus.

## 3. Materials and Methods

### 3.1. Chemicals

Uranyl acetate (UA) was obtained from TAAB Laboratories Equipment Ltd. (Aldermaston, Berks., UK). Optimal cutting temperature (OCT) compound was obtained from Sakura FineTechnical Co. Ltd. (Tokyo, Japan). Carrazzi’s hematoxylin solution and eosin Y ethanol solution were obtained from Wako Pure Chemical Industries Ltd. (Osaka, Japan). Nitric acid (ultrapure analytical reagent) was obtained from Tama Chemicals (Kawasaki, Japan).

### 3.2. Animals and Renal Samples

Renal specimens for determination of elements in situ were obtained from the rats as described [15]. In brief, UA was dissolved in saline, and 0.5 or 2 mg kg^−1^ (body weight) was subcutaneously injected into 10-week-old Wistar male rats (CLEA Japan, Tokyo, Japan). Control rats were injected with saline. Kidneys obtained at 1 and 3 days post-administration collected from three animals per group were analyzed. The Institutional Animal Care and Use Committee at the National Institute of Radiological Sciences approved all animal experiments. The ethic approval numbers are 07-1072-4 (5 March 2011), 16-1029-1 (8 August 2017).

### 3.3. Renal Uranium Concentration

One kidney removed from each rat was divided in half. One half was frozen for elemental imaging, and renal uranium concentrations were determined in a portion of the center area (100 mg) of the other half. Kidney portions were digested with ultrapure nitric acid under microwave heating. Each specimen was diluted with ultrapure water, then uranium concentrations were determined by inductively coupled plasma-mass spectrometry. The limit of uranium detection was 0.015 ng·g^−1^ under our experimental conditions.

### 3.4. Renal Specimen Preparation for Elemental Imaging

Frozen kidney halves cut into 10 µm slices using a cryo-microtome were placed on polypropylene film and dried in a clean box. Serial sections were processed for hematoxylin and eosin staining, or for immunostaining to detect glutamine synthetase (EC 6.3.1.2), which is specific to the S3 segments of the proximal tubule [15].

### 3.5. Imaging of Light Elements in Kidney

The distribution of phosphorus, potassium, and calcium was determined using μ-PIXE analysis using a Model OM-2000 microbeam scanning PIXE system (Oxford Microbeams Ltd., Oxford, UK) with a Si (Li) detector [22]. Elemental images were constructed using the intensity data of the P Kα, and K Kα lines at each point were obtained by scanning the specimens under the following conditions: proton energy, 3.0 MeV; integrated current, 0.2 μC; spatial resolution, 1 μm × 1 μm.

### 3.6. Quantitative Local Analysis of Uranium in the S3 Segments of the Proximal Tubule

Uranium in areas of the S3 segments with and without concentrated phosphorus were quantified by μ-PIXE spot analysis using the μ-PIXE system with a CdTe detector [23]. Briefly, X-ray intensity data of the U Lβ2 were obtained by scanned 1 μm × 1 μm areas of the specimens under conditions of proton energy of 3.0 MeV and integrated current of 0.2 μC with 1 μm × 1 μm spatial resolution. We detected the Lβ2 line for quantitative determination because endogenous renal rubidium [24] interferes with detection of the uranium Lα line. Uranium in microregions was quantified using thin section standards of uranium for microbeam analysis (10 µm; 0–500 μg·g^−1^) [25]. In brief, a calibration line was obtained from the mean total intensity of U Lβ2 in 1 μm × 1 μm areas of three measured points in each standard section.

### 3.7. Uranium Imaging in Kidney

Uranium distribution in the proximal tubules was determined by SR-µXRF using the BL37XU, at SPring-8 (Harima, Japan), using an energy dispersive SR-XRF system with 30 keV monochromatic X-rays [26]. Areas representative the S3 segments of the proximal tubule in the SR-XRF specimen were selected for analysis from corresponding histochemically stained serial sections, and the microbeam (1 μm × 1 μm) was scanned on these areas for uranium imaging. The spatial resolution of the images is described in the figure legends as numbers of steps and distance. The intensity of the uranium Lβ lines (peak width: 16.1–17.6 keV) at each point obtained by scanning the specimens was processed using a personal computer, and two-dimensional elemental maps were created in an 8-bit color scale from 20 μg·g^−1^ (lower detection limit) to the maximum in linear proportion to the elemental concentration. Uranium in microregions was quantified using thin section uranium standards for microbeam analysis (thickness 10 µm; 0–500 μg·g^−1^) [25]. In brief, the calibration line was obtained from the mean total X-ray intensity of uranium Lβ lines in a 1 μm × 1 μm area of 25 measured points in each standard section.

### 3.8. Combination of μ-PIXE, SR-μXRF, and μXAFS

We obtained μXAFS values from renal sections by combining μXAFS with μ-PIXE and SR-µXRF for phosphorus and uranium distribution, respectively, to elucidate the chemical status of uranium in microregions with and without concentrated phosphorus. Phosphorus and potassium distribution was initially determined in renal specimens using μ-PIXE, then uranium was visualized in these specimens using SR-µXRF imaging. Analyzed areas were selected based on the findings on these elemental images. U L_III_-edge μXAFS measurements of concentrated uranium in microregions in the S3 of the proximal tubule were carried out at the BL37XU, SPring-8 [16]. In brief, further uranium imaging was obtained using the X-ray intensity data of the U Lα by scanning the specimens with 17.250 keV X-ray beam. Points with high X-ray intensity were measured using μXAFS, then XAFS spectra were recorded in fluorescence mode with an incident X-ray energy of 17.120–17.250 keV. The XAFS spectrum of UA powder was measured as the standard, and XAFS data were processed using REX2000 software (Rigaku Co., Tokyo, Japan).

## 4. Conclusions

The interaction between uranium and phosphorus is well known in in vitro studies using protein or cultured cells. In the present study, we determined the precise distribution of uranium and phosphorus in rat kidneys using microbeam elemental analysis. The data indicated the colocalization of uranium with phosphorus in in vivo systems after uranium exposure. The elemental composition and uranium chemical status of concentrated uranium varied in the proximal tubules. Moreover, phosphorus localization in the proximal tubules could be one of mechanisms of concentrated uranium formation.

## Abbreviations

μ-PIXE	particle-induced X-ray emission with microprobe
μXAFS	X-ray absorption fine-structure with microprobe
IC	inner cortex
OC	outer cortex
OSOM	outer strips of medulla
SR-μXRF	X-ray fluorescence analysis with microprobe
